# Canonical and Non-Canonical Roles of Human DNA Polymerase η

**DOI:** 10.3390/genes15101271

**Published:** 2024-09-27

**Authors:** Salma Bedaiwi, Anam Usmani, Michael P. Carty

**Affiliations:** DNA Damage Response Laboratory, Centre for Chromosome Biology, School of Biological and Chemical Sciences, University of Galway, Galway H91W2TY, Ireland; s.bedaiwi2@universityofgalway.ie (S.B.); a.usmani2@universityofgalway.ie (A.U.)

**Keywords:** DNA polymerase η, DNA damage tolerance, translesion synthesis, DNA replication, DNA repair

## Abstract

DNA damage tolerance pathways that allow for the completion of replication following fork arrest are critical in maintaining genome stability during cell division. The main DNA damage tolerance pathways include strand switching, replication fork reversal and translesion synthesis (TLS). The TLS pathway is mediated by specialised DNA polymerases that can accommodate altered DNA structures during DNA synthesis, and are important in allowing replication to proceed after fork arrest, preventing fork collapse that can generate more deleterious double-strand breaks in the genome. TLS may occur directly at the fork, or at gaps remaining behind the fork, in the process of post-replication repair. Inactivating mutations in the human *POLH* gene encoding the Y-family DNA polymerase Pol η causes the skin cancer-prone genetic disease xeroderma pigmentosum variant (XPV). Pol η also contributes to chemoresistance during cancer treatment by bypassing DNA lesions induced by anti-cancer drugs including cisplatin. We review the current understanding of the canonical role of Pol η in translesion synthesis following replication arrest, as well as a number of emerging non-canonical roles of the protein in other aspects of DNA metabolism.

## 1. Introduction

DNA damage tolerance plays a key role in maintaining genome stability, by allowing complete replication of damaged DNA without replication fork collapse, thus preventing the formation of double-strand breaks at the sites of fork arrest. DNA damage tolerance pathways include fork reversal and strand switching, error-free homology-based processes that utilise the undamaged complementary nascent strand as a transient template to bypass the damage site [1,2]. The process of translesion synthesis (TLS) is mediated by specialised DNA polymerases that can directly bypass the lesion, based on the capacity of the enzyme active site to accommodate damaged bases, and on the absence of 3′-5′ proofreading exonuclease activity [3,4,5]. However, TLS may be either error-free or error-prone, depending on the combination of lesion type and bypass polymerase involved in a specific TLS event [6,7]. TLS can occur directly at the arrested fork, or at gaps left in the nascent strand following downstream repriming carried out in mammalian cells by the primase activity of PrimPol [8]. The elucidation of the contributions of specific TLS polymerases to each of these scenarios is still a subject of investigation.

The human genome encodes 17 DNA polymerases [9], including the replicative polymerases α, δ, and ε, and a number of specialised polymerases with roles in the replication and repair of damaged DNA [3,6,10]. The key players in TLS include the Y-family polymerases Pol η, ι, and κ, and Rev1, as well as the B-family polymerase Pol ζ [4,6,7,10,11]. PrimPol, which has both TLS and RNA primase activities, contributes to DNA damage tolerance by repriming downstream of sites of replication arrest, leaving a single-strand gap in the nascent strand that is filled post-replication [9,10,11,12,13,14,15,16].

This review focuses on the Y-family polymerase, Pol η, a 78 kDa ubiquitously expressed protein encoded by the *POLH* gene located on human chromosome 6p21. Patients with the skin cancer-prone genetic condition xeroderma pigmentosum variant [17] lack functional Pol η due to mutations in *POLH* (originally called *hRAD30*) [18,19]. In this context, Pol η protects against UV-induced skin cancer by carrying out an error-free bypass of the major UV-induced lesion, the thymine–thymine cyclobutane pyrimidine dimer (TT-CPD). In the absence of Pol η in XPV cells, DNA synthesis is arrested at sites of UV-induced CPDs, and bypasses are eventually carried out by other more-error prone TLS polymerases and mutations accumulate, contributing to skin cancer development [18,19]. While accurately bypassing TT-CPDs is the main role of Pol η in the context of skin cancer protection, Pol η also bypasses a number of other lesions, including, for example, the oxidative lesion 8-oxo-guanine [20]; temozolomide-induced lesions [21]; and cisplatin-induced intrastrand adducts [22,23,24,25]. The capacity of Pol η to bypass platinum-induced lesions is significant for cancer treatment, as the level of Pol η expression can affect the response to widely used platinum-based cancer chemotherapeutics [26,27,28,29,30]. This offers the possibility that modulating Pol η activity using small molecule inhibitors could synergistically enhance the effectiveness of platinum-based cancer chemotherapeutics [1,31,32]. 

**Figure 1:**
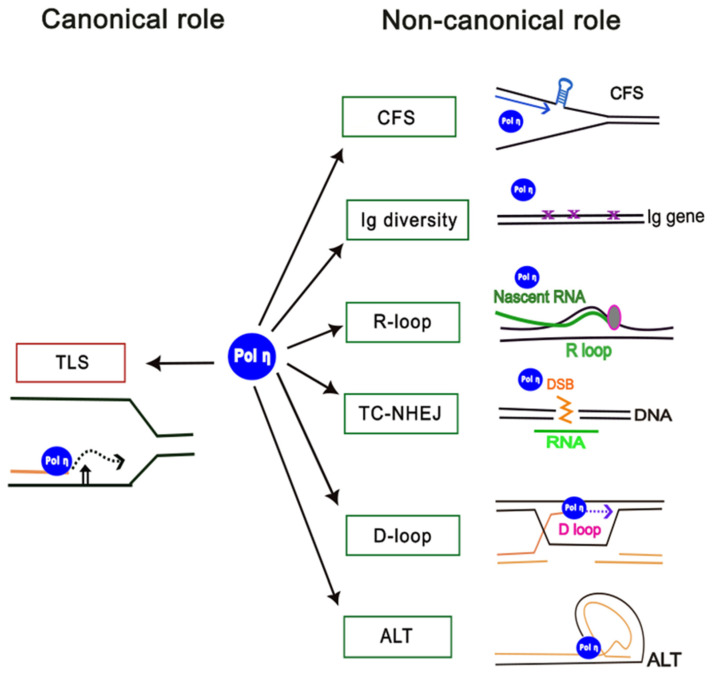
Canonical and non-canonical roles of human DNA polymerase η. The canonical role of Pol η in translesion synthesis (TLS) is shown (left), and non-canonical roles are shown on the right: CFS, replication at common fragile sites; TC-NHEJ, transcription-coupled non-homologous end-joining; Ig diversity, generation of antibody diversity; R-loop, processing of replication at R-loops; D-loop, processing of D-loops; ALT, alternative lengthening of telomeres.

In addition to the canonical role of Pol η in bypassing DNA lesions in the template from exogenous and endogenous sources, there is increasing evidence that Pol η [33], as well as other TLS polymerases [34], plays other non-canonical roles in DNA and RNA metabolism (Figure 1). These non-canonical roles include DNA synthesis at difficult-to-replicate regions of the genome such as common fragile sites [35,36,37,38]; the generation of immunoglobulin diversity during somatic hypermutation (SHM) in memory B cells [39,40,41]; ribonucleotide incorporation and processing of R-loops [42,43,44,45]; reverse transcriptase activity in double-strand break repair by transcription-coupled non-homologous end-joining (TC-NHEJ) [46,47,48]; D-loop extension during homologous recombination [49,50,51], and maintenance of telomere length through the alternate lengthening of telomeres (ALT) pathway [52,53]. Overall, the non-canonical activities of Pol η derive from the capacity of the enzyme active site to accommodate a variety of altered DNA structures; to utilise ribonucleotides in addition to deoxyribonucleotides; and to carry out synthesis using either RNA or DNA as a primer or template. Thus, Pol η mitigates replication stress and promotes genome stability in S-phase cells not only by facilitating bypass of DNA damage-induced lesions in the template strand, but also by carrying out DNA synthesis at difficult-to-replicate regions of the genome that result from altered structures specified by particular DNA sequences. Examples include sequences that form hairpins or G-quadruplexes at common fragile sites [36,38] or telomeric DNA structures [53]. Further, the recent demonstration that human Pol η has reverse transcriptase as well as DNA polymerase activity has expanded its potential cellular roles to include the use of an RNA transcript as a template to facilitate error-free repair of double-strand breaks in transcribed genes in the process of TC-NHEJ [46,48]. Based on the published literature reflecting twenty-five years of research since the first description of human Pol η in 1999 [18,19], this review will consider the canonical role of human Pol η in TLS, and the non-canonical roles of the protein in other areas of DNA and RNA metabolism (Figure 1).

## 2. Structure of Human Pol η

**Figure 2:**
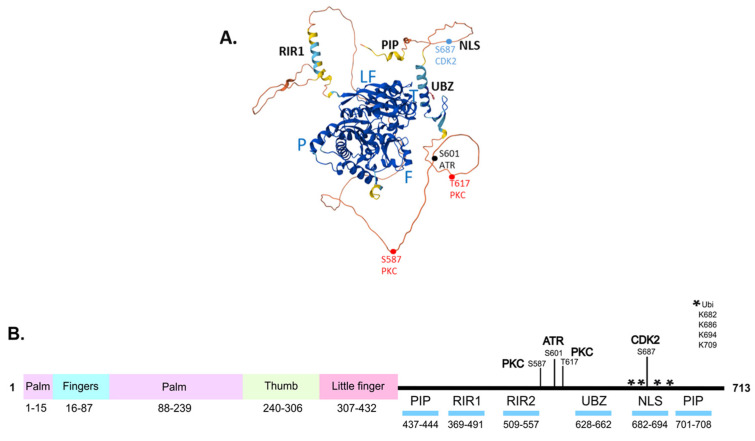
Structure and key domains of human Pol η protein. (**A**). Structural model of human Pol η (UniProt ID Q9Y253) generated using AlphaFold. The positions of the palm (P), fingers (F), thumb (T), and little finger (LF) within the catalytic domain are indicated. PIP, PCNA–interacting peptide; RIR, Rev1–interacting region; UBZ, ubiquitin–binding zinc finger; NLS, nuclear localisation signal. Key phosphorylation sites are shown as dots. (**B**). Schematic diagram of Pol η, showing the positions of the palm, fingers, thumb and little finger within the catalytic domain, and key protein–protein interaction regions in the C–terminus. The location of sites of phosphorylation by PKC, ATR, and CDK2 are indicated; ubiquitination sites are indicated by an asterisk.

Human Pol η is a ubiquitously expressed 78 kDa protein, comprising an N-terminal catalytic domain and a C-terminal protein–protein interaction domain (Figure 2). The catalytic domain includes thumb, finger, and palm domains that are characteristic of other DNA polymerases, as well as the little finger domain (or polymerase-associated domain, PAD) which is unique to Y-family polymerases [4,7]. The co-crystallisation of amino acids 1–432 in the catalytic domain with DNA containing a thymine–thymine CPD [54] or with a guanine–guanine platinum intrastrand adduct [24,55] has provided detailed insight into the structural features of Pol η that allow for efficient bypasses. The protein does not undergo major conformational change during lesion bypass as the more open enzyme active site can accommodate a dinucleotide lesion [55]. Amino acids R61 and S62, which are unique to Pol η, and Q38 facilitate lesion bypass through interactions with the bases of the lesion and with the incoming dNTP [55]. The LF domain, which is not present in other Y-polymerases, provides a local positively charged surface that acts as a molecular splint to maintain the B-DNA structure, counteracting lesion-induced bending of the template which would impede synthesis [7,54]. In addition, the phosphodiester bond formed by Pol η requires three divalent metal ions (Mg^2+^ or Mn^2+^) to bind at the active site, as has been shown using time-resolved X-ray crystallography [56,57,58,59].

The C-terminal domain is largely unstructured but includes several key structural motifs for protein–protein interaction, as well as the NLS (Figure 2). The motifs include the ubiquitin-binding zinc finger (UBZ) motif that mediates interactions with monoubiquitinated PCNA [60], and the Rev1-interacting region (RIR) motif [61] for interactions with Rev1 (Figure 2). The structure prediction programme AlphaFold [62] generates a model of full-length Pol η in which the C-terminal is unstructured, with local α-helical regions consistent with the location of the UBZ and RIR motifs (Figure 2A). Based on the crystallisation of this region, the UBZ consists of two short anti-parallel β-strands comprising amino acids 632–634 and 641–643, and an α-helical region from amino acids 647–662 [60,63]. The UBZ, along with the PCNA-interacting peptide (PIP) motifs [64,65,66], mediate the interaction of Pol η with PCNA, a key step in the recruitment of Pol η to sites of replication arrest. Following replication arrest, PCNA is monoubiquitinated by the Rad6–Rad18 ubiquitin ligase complex [67]. The UBZ binds to K164-monoubiquitinated PCNA, facilitating the polymerase switch that replaces the processive replicative polymerases δ or ε with Pol η [68]. Following lesion bypass, the UBZ binds *in cis* to a number of C-terminal monoubiquitinated sites in Pol η (Figure 2B), competing for binding to ubiquitinated PCNA, leading to release of Pol η from the site of lesion bypass [69]. Pol η also directly interacts with Rad18 via the C-terminal amino acids 556–702, which play a role in targeting the Rad18-Rad6 complex to PCNA at the site of replication arrest, as Rad18 does not directly interact with PCNA [67,70,71]. This is a unique function of Pol η, not shared by other Y-family polymerases. Other interactions, including between Pol η and the pre-mRNA splicing factor SART3, which binds to both Pol η and Rad18, modulate the formation of a complex between Rad18 and Pol η [72]. Interactions with BRCA1 [73] and FANCD2 [74] also promote the recruitment of Pol η to arrested replication forks. 

The structure of the RIR2 motif of Pol η which interacts with the C-terminal domain of Rev1, was determined by Pozhidaeva et al. using solution NMR [61]. The F531–F532 diphenylalanine motif in RIR2, conserved in Y-family polymerases, is critical for Rev1 interaction [61]. Pol η-mediated TLS can also occur independently of PCNA ubiquitination, through this interaction with Rev1, which acts as a scaffold to form a ternary Rev1-PCNA-Pol η complex at sites of replication arrest [8,66,75,76]. The Rev1-dependent recruitment of Pol η to stalled forks is particularly important for ‘on-the-fly’ TLS [8,66,75]. While Rev1 can have a catalytic role in TLS, its major role is to recruit TLS polymerases to the site of DNA damage [76], and to facilitate switching between the inserter DNA polymerase and the extender DNA polymerase pol ζ during lesion bypass [61].

While the majority of protein–protein interactions occur via the C-terminal domain of Pol η, there is evidence that other protein partners, including nucleophosmin [77] and the Werners syndrome (WRN) helicase [78], interact with the N-terminal catalytic core of the protein. Of note, a deficiency in nucleophosmin, which is commonly mutated in AML cells, leads to proteasome-mediated degradation of Pol η [77]. Overall, the evidence indicates that Pol η may exist in sub-complexes with different protein partners that modulate the canonical and non-canonical roles of the protein in TLS or other DNA processing events, as discussed below.

Pol η is post-translationally modified by phosphorylation, ubiquitination, and SUMOylation. Pol η is phosphorylated at a number of sites, including at S587 and T617 by PKC [79], at S687 by CDK2 [80], and at S601 by ATR after DNA damage [80,81,82]. CDK2-mediated phosphorylation at S687 stabilises the protein in the late S and G2 phases of the cell cycle, when Pol η function is required, and facilitates the recruitment of the protein to chromatin [80]. In undamaged cells, Pol η is sequestered by binding to PDIP38, preventing recruitment to the replication fork [82]. Following DNA damage, the Pol η-PDIP38 interaction is disrupted by ATR-mediated phosphorylation of both protein partners. ATR phosphorylates Pol η at S601, promoting interaction between the UBZ domain of Pol η and monoubiquitinated PCNA, facilitating the polymerase switch that replaces Pol δ or ε with Pol η to effect lesion bypass. Following lesion bypass, Pol η S687 is dephosphorylated [80] and the protein is ubiquitinated at a number of lysines in the C-terminal domain by the Pirh2 E3 ligase [69,83]. Pol η now adopts a closed conformation, in which the ubiquitinated domain binds *in cis* to the C-terminal UBZ domain, competing with ubiquitinated PCNA for UBZ binding, thereby releasing Pol η from the site of completed lesion bypass [69]. Pol η is also mono-SUMOylated at K163 by PIAS1 SUMO ligase after TLS, in a Rad18-dependent process that reduces the interaction between Pol η and monoubiquitinated PCNA, leading to STUbL-mediated displacement of Pol η from sites of lesion bypass. Pol η is further poly-SUMOylated at up to 19 other lysine residues that are located within both the catalytic and the C-terminal domains [84]. After being released from the lesion site, Pol η is polyubiquitinated by MDM2, and ultimately targeted for proteasomal degradation [85,86]. In unperturbed cells, K163 mono-SUMOylation is important for the recruitment of Pol η to difficult-to-replicate chromatin regions [87]. Overall, while the regulation of Pol η function during TLS is quite well understood, elucidating the PPIs and PTMs that regulate specific non-canonical functions of Pol η is an important area for future investigation.

While the Pol η protein is ubiquitously expressed, *POLH* mRNA expression is induced following DNA damage in a TP53-dependent manner, mediated by a TP53 response element present in the *POLH* promoter [88,89]. A human papilloma virus 16 (HPV16) infection blocks damage-induced Pol η expression, by HPV E6 protein-mediated TP53 degradation, leading to replication stress and increased sensitivity to chemotherapeutic DNA-damaging agents [90]. *POLH* mRNA is alternatively spliced [91] and polyadenylated [92]. *POLH* mRNA expression is also regulated by the miRNAs miR-93 and miR619, which modulate the sensitivity of ovarian cancer stem cells to platinum-based chemotherapeutic agents [30]. It has recently been reported that the long non-coding RNA taurine upregulated gene 1 (TUG1) up-regulates Pol η levels in ovarian cancer cell lines by binding to miR487-3p and miR-6088, preventing these miRNAs from targeting *POLH* mRNA [93]. TUG1 expression is increased in cisplatin-resistant ovarian cancer, consistent with a role for Pol η in modulating the response to cisplatin treatment [93]. The regulation of Pol η expression by alternative mRNA splicing and by miRNAs in response to different cellular stresses requires further investigation.

## 3. Canonical Role of Pol η

### 3.1. The Canonical Role of Pol η in Translesion Synthesis

While TLS polymerases are considered to be error-prone, TLS protects against the formation of DSBs at sites of prolonged replication fork arrest, and therefore contributes to genome stability. The canonical role of Pol η is in bypassing lesions that block genomic DNA replication by DNA polymerases δ and ε [5]. Structural studies [7,54,55], lesion bypass assays using purified Pol η protein in vitro, and analysis of bypass following transfection of cells with individual lesions in plasmid constructs [94], demonstrate that Pol η can bypass a variety of lesions, including UV-induced CPDs [95], 8-oxoG adducts [96,97], O6-methylguanine [98], cisplatin-induced intrastrand adducts [24,99], temozolomide-induced alkylation damage [21], misincorporated 5′-fluorouracil (5′FU) residues [100], and bulky adducts such as dG-N^2^-(+)-trans-*anti*-benzo[a]pyrene [101] and dG-N^2^-IQ [2-amino-3-methylimidazo[4,5-f]quinolone] [102]. Pol η also bypasses a non-templating abasic (AP) site, with the addition of a purine nucleotide [103,104,105]. Depending on the type of lesion, bypass may be error-free or error-prone [63]. In the case of UV-induced DNA damage, while Pol η can bypass thymine–thymine CPDs accurately [95], it cannot bypass a (6-4)PP [106]. Complete bypass requires the combined action of a second TLS polymerase such as Pol ζ, Pol ι, or Pol θ, in a two-polymerase mechanism of lesion bypass [95,106,107]. In this process, Pol η is limited to inserting a single nucleotide opposite the 3′T of a (6-4)PP, with the completion of the bypass requiring the activity of the second TLS polymerase. Although UV-induced (6-4)PPs are less frequent in the genome than CPDs, this lesion is highly mutagenic. Compared to *cis-syn* thymine-thymine CPDs, (6-4) PPs cause a more marked structural distortion, placing the 3′T perpendicular to the 5′T in the lesion [106]. Pol η inserts a guanine rather than an adenine opposite the 3′T of a thymine–thymine (6-4)PP, as a hydrogen bond cannot be formed between the carbonyl oxygen of the 3′T and the amino group of an incoming adenine residue, while hydrogen bond formation is possible with a guanine residue [106]. Pol ζ then adds an adenine opposite the 5′T and extends from the primer terminus to complete bypass of the (6-4)PP, generating a mutation at the 3′T of the lesion [106].

XPV cells which lack functional Pol η are hypermutable after UV-irradiation [108] as a result of error-prone bypass of CPDs by other TLS polymerases including Pol ι and Pol ζ [5]. The consequences of Pol η-deficiency for UV-induced mutagenesis were recently reported based on whole-genome sequencing of 14 skin tumour samples from XPV patients [109]. The frequency of mutations in the genome was increased three-fold in XPV-derived skin tumours compared to sporadic skin tumours, indicating that Pol η normally suppresses mutagenesis [109]. The frequency of mutations at thymine–thymine sites was higher in XPV-derived tumours, and the majority of mutations at dipyrimidine sites were at the 3′ nucleotide, supporting the two-polymerase model of UV-induced lesion bypass in the absence of Pol η [109]. Of interest, the mutation spectrum in the genome of skin tumours from XPV patients also includes mutations of the purine in a T-A/G [109] or C-A sequence context [110]. This may reflect a role for Pol η in bypass of an as-yet unidentified UV-induced purine lesion [109,110]. As cytosine-containing dimers, rather than thymine-thymine dimers, are the major source of UV-induced mutations in skin cancers in the non-XPV population [109,111], it has been proposed that Pol η activity may lead to mutations by accurately bypassing deaminated cytosine bases within C-containing CPDs [111]. The deamination of cytosine and 5-methyl-cytosine generates uracil and thymine, respectively. Since the rate of cytosine deamination is also increased in the context of a CPD [111], accurate bypass of the deaminated bases by Pol η could account in part for the high frequency of C->T transition mutations characteristic of UV-induced mutagenesis [111].

The capacity of Pol η to bypass cisplatin-induced intrastrand crosslinks is of significant interest given the importance of platinum-based drugs as mainstays of cancer chemotherapy. Lesion bypass is a potential target for the development of inhibitors that could enhance the effectiveness of platinum-based drugs [1]. Cisplatin induces DNA intrastrand purine–purine adducts, as well as interstrand crosslinks (ICLs). Considerable evidence supports a role for Pol η in bypassing cisplatin-induced intrastrand adducts, consistent with the fact that Pol η has a sufficiently large active site to accommodate and bypass these lesions [24,55,112]. Purified Pol η can bypass cisplatin adducts in oligonucleotide templates in vitro [99]. X-ray crystallographic studies have provided detailed insight into how the active site of human Pol η accommodates a guanine–guanine intrastrand adduct, and how structural changes in the protein upon lesion binding facilitate lesion bypass [24,55]. Gln 38, Arg 61, and Ser 62 play key roles in the incorporation of nucleotides opposite the guanine–guanine adduct [55], while the LF domain loop Q373-379 makes contact with the major groove and shifts following nucleotide incorporation to allow extension to be completed [24]. In vivo, Pol η-deficient XPV cells are more sensitive to cisplatin compared to wild-type cells [26,27], and Pol η expression modulates nascent strand length in cisplatin-treated XPV cells [25]. Consistent with a functional role for Pol η in the response of cancer cells to cisplatin, miRNA-mediated down-regulation of Pol η increases the sensitivity of ovarian cancer stem cells to cisplatin [30]. Recent evidence that Pol η plays a role in bypass of adducts induced by other chemotherapeutic drugs including temozolomide [21] and 5′fluorouracil [100] widens the repertoire of lesions induced by cancer chemotherapeutics that can be bypassed by Pol η. In the case of 5-FU, it is notable that Pol η can both incorporate 5-FU into DNA as well as bypass the resulting 5-FU lesions, implicating Pol η activity in the response to 5-FU-based drug regimens used in the treatment of colorectal cancer [100]. Interestingly, Pol η also plays a role in the response to the topoisomerase inhibitor etoposide, through a non-canonical function in the repair of double-strand breaks by non-homologous end-joining (see below) [113].

### 3.2. Cellular DNA Damage Tolerance Pathways

**Figure 3:**
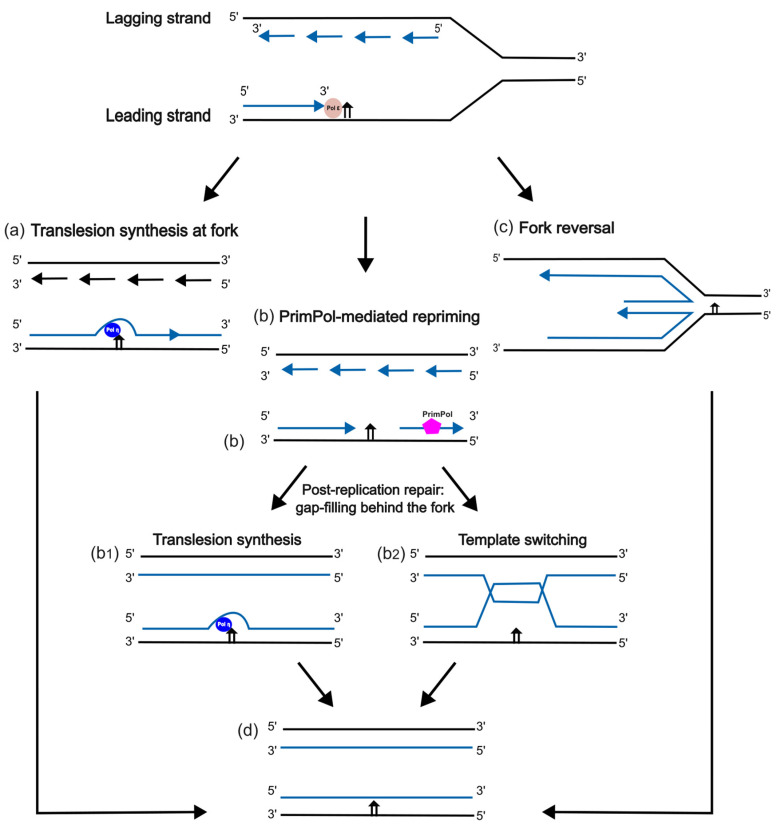
Schematic diagram of DNA damage tolerance pathways. (**a**) TLS by Pol η at the replication fork; (**b**) post-replication gap filling by TLS (**b1**), or by template switching (**b2**), following PrimPol-mediated repriming; (**c**) Pol η-independent fork reversal; (**d**) completed bypass, showing a lesion still present in the template strand.

TLS by Pol η or other TLS polymerases takes place in the context of cellular DNA damage tolerance pathways that stabilise the replication fork and allow replication to continue on the damaged strand, preventing fork collapse to maintain genome stability [114,115], as shown in Figure 3. These pathways include fork reversal and template switching; TLS in order to directly bypass the lesion at the fork; and repriming downstream of the lesion with the generation of single-stranded gaps that are subsequently filled by the action of TLS polymerases working behind the fork, or by a template switching mechanism (Figure 2). The selection of a particular pathway is dictated by the activity of pathway-associated proteins, as well as by the type and level of the DNA damage [116]. The replicative polymerases, Pol ε on the leading strand and Pol δ on the lagging strand, generally cannot bypass lesions in the template, resulting in the replication fork slowing or stalling [114]. However, blocking replication on one strand does not necessarily affect DNA synthesis on the other strand but instead can lead to fork uncoupling [114]. Further, a lesion on the lagging strand does not interfere with replication fork progression to the same extent as a lesion on the leading strand, as another Okazaki fragment can be initiated by Pol α-primase upstream of the damage, allowing continued replication fork progression [114,117].

Post-translational modification of PCNA plays a role in the choice of the damage tolerance pathways shown in Figure 2. As outlined above, the RAD6/RAD18 complex, consisting of the E2 ubiquitin-conjugating enzyme RAD6 and the E3 RING-finger ubiquitin ligase RAD18, is recruited to sites of replication arrest by interacting with RPA molecules that coat regions of single-stranded DNA generated on the parental strand. RAD6/RAD18 monoubiquitinates PCNA on K164, which promotes the recruitment of Pol η to the site of replication arrest through interaction of the C-terminal UBZ domain (Figure 1) with the ubiquitin moiety of PCNA [67]. The interaction of Pol η with PCNA through the PIP domain [64,65] and with Rev1 through the RIR domain is also required for efficient recruitment of Pol η to sites of replication arrest [8,61,75].

Alternatively, to promote error-free bypass by fork reversal (Figure 3c), PCNA can be polyubiquitinated through the formation of K63-linked multi-ubiquitin chains by the E2 complex Mms2-Ubc13, and by HLTF or SHPRH, human homologs of the yeast E3 ligase RAD5 [116,118,119,120]. In response to UV-induced damage or other bulky lesions, HLTF binds to the primer terminus at arrested forks [121,122,123]. Following HLTF-mediated PCNA polyubiquitination, the DNA translocase ZRANB3 binds to the modified PCNA to promote fork reversal and restart replication [124]. As well as having ubiquitin ligase activity, HLTF can also catalyse this fork reversal step [122]. In human HLTF-knockout cells lacking HLTF-mediated fork reversal, replication fork progression still continues after UV irradiation, consistent with TLS (Figure 3a) and PrimPol-mediated repriming (Figure 3b) acting to bypass the damage [125]. Conversely, the recruitment of HLTF suppresses other DDT pathways [126], indicating that there is competition between different DDT pathways at the sites of replication arrest. Fork reversal allows error-free bypass of lesions by providing the undamaged complementary nascent strand as a transient template for replication (Figure 3c). However, this process also generates single-ended DSBs (seDSBs) which are vulnerable to degradation by Mre11 and other nucleases [2,127]. BRCA1 and BRCA2 proteins play key roles in protecting seDSBs from Mre11-mediated DNA degradation [2,128,129,130]. Other DDT pathways, including TLS, are therefore important to ensure lesion bypass and fork stability in BRCA-deficient cancer cells [131], with potential opportunities for the development of novel targeted cancer therapies [2].

Pol η-mediated TLS occurs either directly at the fork (Figure 3a) or during the filling of post-replicative gaps (Figure 3b). Pol η has been shown to directly bypass UV-induced CPDs and cisplatin lesions at the fork (Figure 3a) in human cells [25,116,132,133,134], and in a reconstituted yeast TLS system in vitro [135]. Pol η is associated with the ongoing replication fork in normal cells and is recruited to sites of replication arrest [87]. Direct TLS at the fork promotes genome stability by allowing replication to continue without the generation of ssDNA gaps or strand breaks [136]. After the completion of lesion bypass, Pol η is post-translationally modified by ubiquitination and SUMOylation, as described above, leading to the release of Pol η and replacement with pol δ, which extends the nascent DNA strand until it reaches the Cdc45-MCM-GINS-Pol ε-engaged (CMGE) complex, where Pol ε continues the replication of the leading strand [137]. The lesion remains in the DNA template and may be subsequently repaired by NER or BER.

If a lesion is not bypassed directly by TLS at the fork (Figure 3a), an alternative pathway is repriming, in which a primase synthesises a new RNA primer downstream of a lesion on the leading strand. This leaves behind a single-stranded DNA (ssDNA) gap in the nascent strand while allowing replication to continue (Figure 3b). Initial evidence from yeast demonstrated that repriming was carried out by Pol α-primase, leading to an accumulation of ssDNA gaps, which was increased in *rev1*-, *rev3*-, or *rad30*-defective strains [138]. In human cells, the RNA primase activity of PrimPol rather than Pol α-primase plays the central role in repriming, in particular following replication arrest at lesions on the leading strand [8,15,139,140] (Figure 3b). Gaps accumulate behind the replication fork as the replisome continues to synthesise DNA beyond the lesions [117,140,141,142,143,144]. Gap filling then becomes a critical process as unrepaired single-strand gaps can be converted to more lethal double-strand breaks by nuclease action [2,134,136]. Consistent with a role for Pol η-mediated TLS in preventing the conversion of ssDNA gaps into double-strand breaks, the DSB marker γH2AX co-localises with post-replicative ssDNA gaps in UV irradiated Pol η-deficient cells [134,145].

Post-replicative ssDNA gaps are filled either by TLS by Pol η [145] or other TLS polymerases, in particular Pol ζ [8,117,146,147,148] (Figure 3(b1)), or by template switching (Figure 3(b2)) in the late-S or G2 phases of the cell cycle [116,149]. The mode of the interaction of PrimPol with RPA molecules bound to the ssDNA positions PrimPol downstream of RPA, and defines the length of the gap [140]. It was recently demonstrated that, in TK6 lymphoblastoid cells, PrimPol is recruited soon after replication arrest, restricting the length of the ssDNA gaps that are generated and preventing the formation of long stretches of ssDNA around the lesion site [8]. Repriming by PrimPol thus plays a key role in ensuring efficient co-ordination between TLS at the fork and at post-replicative gaps [8]. Post-replicative gaps can also be repaired in an error-free manner by template switching (Figure 3(b2)), initiated by RAD51-mediated strand invasion, where the undamaged nascent strand from the sister chromatid serves as a transient replication template to facilitate error-free lesion bypass [150].

The process of post-replicative gap filling, also termed gap suppression, ensures that single-strand gaps are filled before cells enter mitosis. This process is of increasing interest as such gaps can also arise as a result of oncogene-induced replication stress, or in cells where other tolerance pathways are compromised such as in *BRCA*-deficient cells [136]. Targeting the process of post-replicative gap-filling may therefore have therapeutic potential [136], and a more detailed understanding of the molecular mechanisms of post-replicative gap filling is important in this context [67,151].

## 4. Non-Canonical Roles of Pol η in Replication and Repair

In addition to its’ established role in translesion synthesis described above, there is increasing evidence that Pol η plays a number of other non-canonical roles in DNA metabolism [33] (Figure 1).

### 4.1. Replication at Common Fragile Sites (CFS)

Regions of repetitive sequences in the genome present a challenge to the DNA replication machinery, resulting in increased susceptibility of such genomic regions to DNA breakage [152,153,154]. Such sites, termed common fragile sites (CFSs), often adopt non-B DNA conformation, forming hairpins or G-quadruplex (G4) structures that are a barrier to replication [153,155]. While replicative polymerases including Pol ε cannot carry out efficient synthesis through repetitive sequences and G4 structures, Pol η retains the ability to traverse these templates [156,157]. Pol η-deficient cells show defects in the replication of CFSs, supporting a role for Pol η in the replication of difficult-to-replicate genomic regions [35,36,38,157,158,159]. Pol η also plays a role during replication stress induced by Myc-overexpression [160] or dNTP depletion [87,157]. Consistent with roles in replication even in the absence of DNA lesions in the template, Pol η is found to be associated with replication forks in undamaged cells [87,161]. The recruitment of human Pol η to CFSs appears to be independent of interactions with PCNA [157] but requires Rad18-dependent SUMOylation of Pol η on K163 by the SUMO ligase PIAS1 [87]. Rad18 binds to both Pol η and PIAS1, acting as a scaffold to promote K163 SUMOylation [87]. The expression of the K163R-mutated form of Pol η, which cannot be SUMOylated, leads to the generation of under-replicated DNA, and segregation defects in mitotic cells [87]. Phosphorylation of Pol η at S687 by CDK2 in the late S and G2 phases [80,82,162] may also contribute to the recruitment of Pol η protein to chromatin for CFS replication which occurs in the late S phase [35]. XPV cells lacking Pol η show increased genomic instability at a number of CFS loci, including FRA16D and NEGR1, with the formation of micronuclei containing NEGR1 sequences, consistent with incomplete replication of this CFS region in the absence of Pol η [38]. Corradi et al. [110] further reported that the frequency of mobile element insertion and retrotransposition was increased in a cohort of 11 skin tumours from Pol η-deficient XPV patients, indicating that Pol η normally suppresses such genomic rearrangements, potentially by reducing strand break formation at sites that could act as entry points for mobile elements. Notably, the replication of CFSs by Pol η may contribute to sequence variation at these regions in the general population, as genomic DNA sequencing revealed a Pol η-specific mutation signature within the sequenced CFS regions [38]. Overall, Pol η contributes to genomic stability by ensuring the efficient replication of difficult-to-replicate genomic regions, in addition to its’ established role in TLS at sites of DNA damage.

### 4.2. Generation of Immunoglobulin Diversity

The non-canonical role of Pol η in the generation of immunoglobulin diversity during somatic hypermutation (SHM) of immunoglobulin (Ig) genes is well-established [39,40,41,163,164,165,166]. Pol η therefore contributes significantly to the adaptive immune response that generates an array of antibodies to combat diverse pathogens [39]. Pol η expression is strongly induced in activated B cells in germinal centres where SHM occurs, compared to resting B cells [39,41], and is thus available for error-prone DNA synthesis that generates characteristic mutations at A/T base pairs. During SHM, APOBEC-mediated cytosine deamination generates uracil in the genome [5]. Mutations at G/C base pairs are not dependent on Pol η but result from the replication of uracil-containing DNA by polymerases δ and ε [5]. However, evidence from cell-based systems and from knockout mouse models have clearly demonstrated that mutations at A/T base pairs in Ig genes during SHM are strongly Pol η-dependent [39,40,41,163,164,165,166]. Pol η is recruited to sites of SHM through interaction with PCNA [167,168,169], and generates characteristic WA to WG mutations as a result of efficient misincorporation of dGTP opposite the thymine of an A/T base pair in the WA sequence context [170] during error-prone DNA synthesis at single-strand gaps, generated by the action of the Msh2/Msh6 mismatch repair complex to remove uracils from DNA [5,40]. Consistent with the role of Pol η in SHM, the frequency of mutations at A/T bases in Ig genes in memory B cells is reduced in XPV cells [41]. In *POLH*^−/−^-knockout mice, there is an increased frequency of transversion mutations in Ig genes, consistent with a role for Pol κ in SHM when Pol η in not available [165]. Of note, it was recently reported that, in two cohorts of XPV patients, the absence of Pol η also leads to an age-dependent increase in the frequency of 10–20 base-pair deletions at the J_H_4 intron junctions of Ig genes [171]. In the absence of Pol η, it is proposed that an alternate polymerase, possibly Pol θ, generates deletions during SHM [171]. This indicates a dynamic interplay between TLS polymerase activity, replicative stress, and genome stability in memory B cells.

The A/T base pair-specific mutational signature in Ig genes, observed under conditions of increased Pol η expression and replicative stress, is referred to as signature 9 or SBS9 [172]. While SHM is targeted to Ig genes, one consequence of elevated Pol expression in memory B cells is that the enzyme potentially has access to other regions of the genome. Consistent with this, the Pol η-associated SBS9 signature can be identified across the genome of memory B cells [172]. Thus, Pol η may contribute to the frequency of background mutations in cancer-related genes, as well as to somatic hypermutation in Ig genes, with consequences for lymphoma development [172]. Furthermore, the SBS9 mutational signature has also been identified in various other cancers, including pancreatic tumours [38,173]. This highlights the broader implications of increased Pol η expression for cancer development, indicating the potential link between Pol η-mediated mutagenesis and tumorigenesis.

### 4.3. R-Loop Processing, and Ribonucleotide Incorporation

As well as carrying out DNA synthesis on damaged and undamaged templates as outlined above, there is increasing evidence that Pol η can (i) use an RNA strand as a primer for DNA synthesis [44,45,46]; (ii) incorporate ribonucleotides into DNA, including during lesion bypass [42,43]; (iii) synthesise RNA and carry out transcriptional bypass of lesions [174,175,176]; and (iv) generate a DNA copy of an RNA template by acting as a reverse transcriptase [46,48]. Consistent with this broader role for Pol η, the protein binds to DNA-DNA, DNA-RNA, and RNA-DNA templates with approximately equal affinities [46].

R-loops, formed when a nascent RNA molecule hybridises with double-stranded DNA creating a three-stranded structure consisting of an RNA-DNA hybrid and a region of single-stranded DNA, contribute to genome instability [177,178]. Pol η can utilise the RNA strand in an R-loop structure as a primer to initiate DNA synthesis in vitro, an activity that could facilitate fork restart after replication arrest [44]. Purified Pol η has also been shown to utilise an RNA primer to bypass a CPD or an 8-oxoG lesion in a DNA template [46], and could therefore play a role in lesion bypass during replication initiation, or at an Okazaki fragment on the lagging strand [46].

While Pol η shows strong selectivity for incorporation of dNTPs rather than ribonucleotides, Pol η can nonetheless incorporate ribonucleotides during translesion synthesis past CPDs and 8-oxoG lesions [42], as well as opposite guanine–guanine intrastrand cisplatin adducts [43]. X-ray crystallographic studies show that the position of the ribose sugar in the active site is altered compared to the deoxyribose in a dNTP, due to the presence of the F18 residue in the active site which acts as a steric gate [42]. Time-resolved X-ray crystallography of extension of a ribonucleotide primer by Pol η demonstrated that the presence of the 2′OH of the ribosugar increased the misincorporation frequency during extension from the ribonucleotide end [45]. Under conditions of hydroxyurea-induced dNTP depletion In vivo, the incorporation of ribonucleotides allows yeast Pol η to continue DNA synthesis even when dNTP levels are limiting, ensuring the completion of DNA replication [179]. Under these conditions, RNaseH activity is critical to remove the incorporated ribonucleotides from the genome, to prevent formation of single-strand breaks at these sites [179]. It has also been proposed that Pol η could facilitate elongation of a blocked RNA strand during transcription, by virtue of the capacity to both incorporate ribonucleotides opposite a DNA lesion and extend the ribonucleotide strand [179]. In support of this role, it has been shown that purified Pol η can bypass lesions in an in vitro transcription system [175,176]. While further investigation is required, this expands the potential roles of Pol η in the cell to include RNA as well as DNA synthesis.

### 4.4. Reverse Transcriptase Activity in TC-NHEJ

Recent evidence that Pol η not only has DNA polymerase activity but can also act as a *bona fide* reverse transcriptase, using RNA as a template for the synthesis of a complementary DNA strand, has further expanded our understanding of the non-canonical roles of Pol η in the cell [46,48,180]. Consistent with this role of Pol η In vivo, the depletion of Pol η in HEK293 cells, or a lack of Pol η in XPV cells, reduces reverse transcriptase activity in cell extracts, which is restored by the addition of purified Pol η [46,47,48]. The RT activity of Pol η is proposed to play a central role in the process of transcription-coupled non-homologous end joining (TC-NHEJ), where an RNA transcript provides a complementary template for error-free repair of double-strand breaks in transcribed genes [33,46,48]. This process is important to prevent the accumulation of mutations in coding regions of the genome in non-proliferating cells, which cannot utilise homologous recombination for error-free double-strand break repair [48]. Mechanistic evidence supports a role for Pol η in TC-NHEJ. Human Pol η is found associated with nascent RNA, and as part of a multiprotein complex with RNA Pol II in HEK293 cells [48]. Pol η depletion leads to an increase in R-loop formation in the genome after treatment with bleomycin, indicating that RNA is normally used as a template to fill the gap at DSB sites via the reverse transcriptase activity of Pol η [48]. The N-terminal of Pol η also interacts with the NHEJ scaffold protein Kruppel-associated box-associated protein 1 (Kap1) forming a ternary complex with Rad18 during repair of etoposide-induced DSBs [113], consistent with the proposed role for Pol η in double-strand break repair by TC-NHEJ.

### 4.5. D-Loop Extension Activity during Homologous Recombination

Unlike other DNA polymerases, Pol η can carry out the extension of a D-loop [49], a three-stranded DNA intermediate formed during HR [181]. In vitro studies have demonstrated that recombinant Pol η can recognise and preferentially bind to D-loop structures, efficiently extending the invading DNA strand [49,50]. Moreover, Pol η-deficient XPV cells are defective in D-loop extension, supporting the role of Pol η in this step of HR [49]. D-loop extension by Pol η is PCNA-independent as the PIP box is dispensable [50]. However, interaction of PALB2 and BRCA2 with Pol η stimulates extension of the invading strand during HR-mediated repair at collapsed replication forks [51]. This function of Pol η may be important in break-induced replication (BIR), required to repair a single-ended DSB formed by template strand breakage at the site of replication fork collapse [2]. Overall, the ability of Pol η to carry out D-loop extension highlights a role in repair of replication arrest-induced strand breaks by HR, separate from the canonical role in TLS.

### 4.6. Alternate Lengthening of Telomeres

The maintenance of telomere length at the ends of chromosomes, either by re-expression of telomerase [182], or by using the alternate lengthening of telomeres (ALT) pathway [183], is crucial for the survival of cancer cells. Consistent with a role for Pol η in telomere replication, XPV cells show an increased frequency of damage-induced telomere aberrations [52]. Pol η is localised to telomeres after UV-induced DNA damage, reducing ATR-dependent damage response signalling [52]. In the absence of damage, Pol η is required for DNA synthesis during ALT [53]. Pol η interacts with TRF1, a component of the shelterin complex that normally protects telomeres from the action of DNA repair proteins [53]. Analogous to its role in DNA loop extension, Pol η initiates recombination-mediated DNA synthesis on telomeric DNA, generating DNA strands that are subsequently extended by Pol δ [53]. Reduced Pol η levels lead to telomere aberrations, including exchange of DNA between telomeres, and extensive telomeric DNA synthesis by Pol δ in mitosis [53]. Thus, Pol η plays a role in ensuring that telomere structure is maintained during replication in cancer cells, with implications for cancer cell survival.

## 5. Pol η Mutations and Cancer

Given the key role of Pol η in TLS, there is increased interest in targeting TLS polymerases, including Pol η, in cancer cells, to enhance the effectiveness of chemotherapeutic DNA damaging agents, particularly in genetic backgrounds that make cancer cells more dependent on TLS for survival [1,10]. However, given the non-canonical roles of Pol η in a number of other processes outlined above, the wider implications of targeting Pol η in the cell must be considered [33], but these pathways could also provide new opportunities for precisely targeted interventions [48].

The role of Pol η in suppressing skin cancer development is clear, since inactivating mutations in Pol η lead to XPV, as result of increased mutagenesis during replication of UV-damaged DNA in skin cells [18,19,108,109,184]. The majority of *POLH* mutations in XPV are either missense mutations altering key residues that inactivate protein function or frameshifts leading to protein truncation [108,184]. The inactivating missense mutations mainly occur in the N-terminal catalytic domain [108,185,186,187]. One missense mutation, T692A, in the C-terminal domain of Pol η has been reported in XPV [188]. This mutation leads to the formation of a 721 amino acid protein, due to the presence of a second point mutation that eliminates the normal stop codon, causing the addition of eight amino acids to the C-terminal of Pol η [188]. The resulting protein has near normal lesion bypass activity in vitro but is unstable in cells due to proteasomal degradation of the altered protein [188]. Splicing mutations in *POLH* that lead to loss of Pol η protein expression have also been reported in a small number XPV patients [41,189,190]. Of note, in two cases the causative splicing mutations are located at splice junctions within or upstream of the untranslated exon 1 [41,189].

In addition to inactivating mutations in *POLH* that cause XPV disease, single-nucleotide polymorphisms that encode missense variants of Pol η have been identified in the non-XPV population [191,192]. However, the functional effects of most of these changes on Pol η protein function have not been investigated to date. Yeom et al. [193] recently reported analysis of a series of germline missense variants in Pol η. Purified Pol η proteins carrying either the C34W, I147N, or R167Q missense mutations showed a reduced ability to bypass cisplatin lesions in vitro, and these missense variants could not fully complement cisplatin sensitivity when expressed in Pol η-deficient cells [193]. This provides evidence that polymorphisms in *POLH* can affect Pol η activity. The *POLH* genotype could be important in the response of tumours to DNA damaging cancer chemotherapeutics [194], particularly as cancer genomics databases (COSMIC; cBioportal) list somatic *POLH* mutations that have been identified by sequencing genomic DNA from tumour tissues. Given the non-canonical roles of Pol η, it will be important to understand how individual mutations affect the function of the protein not only in TLS but also in other roles, including replication of regions of undamaged DNA, somatic hypermutation, and in TC-NHEJ. Specific mutations could also affect key protein–protein interaction domains, or sites of post-translational modification, rather than directly affecting DNA or RNA synthesis activity [184]. Further, *POLH* mRNA is over-expressed in a subset of cancers, including non-small cell lung cancer and head and neck squamous cell carcinoma, as a result of amplification of the *POLH* genomic locus on chromosome 6 [29,159,194]. Over-expression of Pol η has consequences for genome stability, for example in memory B cells [172], and in tumours where the Pol η-dependent SBS9 mutation signature can be detected [38,173].

As noted above, inhibitors of Pol η are under investigation in order to enhance the effectiveness of DNA damaging agents in chemotherapy [32]. A number of inhibitors of Pol η and other TLS polymerases have been identified [1,31,32,195]. Inhibition may be more effective depending on the status of other DNA damage tolerance pathways in the tumour [10,196]. Given the non-canonical roles of Pol η in addition to TLS outlined here, the effects of inhibition on these activities should also be investigated, to better understand the consequences of such inhibitors for genomic stability.

## 6. Conclusions and Future Perspectives

Pol η has key roles in bypassing lesions, and contributes to both accurate and mutagenic DNA replication, depending on the lesion. Its’ major role is in bypass of UV-induced lesions, as evidenced by the consequences of *POLH* mutations in XPV. In the case of UV-induced lesions, Pol η bypass reduces the frequency of mutations at dipyrimidine sites, exemplified by the increased mutation frequency in XPV-derived cells and skin tumours. Overall, TLS by Pol η ensures continued nascent strand synthesis, contributing to cell survival, albeit at the cost of introducing mutations to the genome during bypass of certain lesions. Because the active site of Pol η can accommodate ribonucleotides as well as deoxyribonucleotides, and carry out synthesis using RNA as well as DNA as the primer or template, there is increasing evidence that Pol η also plays key roles in a number of other cellular processes, including replication of common fragile sites, generation of immunoglobulin gene diversity, reverse transcription in TC-NHEJ, and primer extension during recombinational repair and telomere maintenance. It will be important to better understand the relative importance of the canonical and non-canonical activities of Pol η, including how access of Pol η to replication and repair sites is regulated in different chromosomal contexts. In addition, the PPIs and PTMs that regulate specific non-canonical functions are not well understood. The effects of mutations in Pol η, and the effects of Pol η inhibitors on these functions also need to be investigated. These insights will be important to further advance targeting of Pol η to enhance the effectiveness of DNA damage-based cancer therapies, and to understand the consequences of mutations in *POLH* for genome stability.

## Data Availability

Data sharing is not applicable to this review article.
